# Effect of Static Magnetic Field Assisted Thawing on Physicochemical Quality and Microstructure of Frozen Beef Tenderloin

**DOI:** 10.3389/fnut.2022.914373

**Published:** 2022-05-24

**Authors:** Junbo Jiang, Liyuan Zhang, Jianbo Yao, Yue Cheng, Zhongrong Chen, Gang Zhao

**Affiliations:** ^1^Research and Engineering Center of Biomedical Materials, School of Biomedical Engineering, Anhui Medical University, Hefei, China; ^2^School of Basic Medicine, Anhui Medical University, Hefei, China; ^3^College of Life Sciences, Anhui Medical University, Hefei, China; ^4^Department of Electronic Science and Technology, University of Science and Technology of China, Hefei, China

**Keywords:** static magnetic field, thawing, physicochemical quality, microstructure, beef tenderloin

## Abstract

Although freezing is the most common and widespread way to preserve food for a long time, the accumulation of microstructural damage caused by ice crystal formation during freezing and recrystallization phenomena during thawing tends to degrade the quality of the product. Thus, the side effects of the above processes should be avoided as much as possible. To evaluate the effect of different magnetic field strength assisted thawing (MAT) on beef quality, the indicators associated with quality of MAT-treated (10–50 Gs) samples and samples thawed without an external magnetic field were compared. Results indicated that the thawing time was reduced by 21.5–40% after applying MAT. Meat quality results demonstrated that at appropriate magnetic field strengths thawing loss, TBARS values, cooking loss, and shear force were significantly decreased. Moreover, by protecting the microstructure of the muscle, MAT significantly increased the a^∗^ value and protein content. MAT treatment significantly improved the thawing efficiency and quality of frozen beef, indicating its promising application in frozen meat thawing.

## Introduction

In recent years, with the explosive growth of population, various global food security issues have become increasingly severe. Besides increasing food production, reducing food waste and losses is also significant to solving the problem of food shortage. Improvements in food freezing and thawing technology are key to reducing food loss and waste (1). Freezing is the most common and widespread way to preserve food for a long time and plays an important role in the food field (2, 3). In the meat industry, it is also a guarantee to ensure that products can be safely supplied to all parts of the world (4). In this situation, meat is usually in a frozen state prior to purchase. Naturally, frozen meat needs to be thawed prior to most subsequent processing and cooking (5, 6). Although freezing can maintain the taste and quality of food in the long term, the accumulation of microstructural damage caused by ice crystal formation during freezing and recrystallization phenomena during thawing tends to degrade the quality of the product (7, 8). During the thawing process, a heat absorption reaction occurs, which breaks the hydrogen bonds in the ice crystals. Occasional recrystallization of the freshly thawed layer may occur due to the endothermic reaction of the inner layer if the heat exchange is inefficient (9, 10). At the same time, thawing always induces quality losses in frozen meat such as thawing loss, color deterioration, texture changes, and loss of nutrients (11, 12). The degree of quality loss is determined by numerous factors, including preparation before freezing, storage temperature, thawing rate, especially the thawing method (5, 13). Therefore, it is essential to discover and optimize appropriate methods and circumstances for thawing meat.

Currently, water thawing and air thawing at room temperature are the most commonly applied traditional thawing methods. However, these methods are time-consuming and the meat quality may be deteriorated during the process of defrosting (10, 14). Therefore, thawing technologies such as ultrahigh-pressure, microwave, and high-voltage electrostatic field have emerged in recent years. Although these emerging thawing technologies can significantly accelerate the defrosting of meat, they still have certain shortcomings (8). Expensive equipment is needed for ultrahigh-pressure, so the cost of this method is relatively high (15). Microwave thawing tends to cause uneven heating, which makes it difficult to control the quality of the product. In addition, this method cannot accommodate metal packaging (16, 17). For high-voltage electrostatic field thawing, its output voltage is high, which poses a safety hazard (11). These methods are limited by these shortcomings and cannot be used in certain scenarios.

Magnetic field is an environmentally friendly technique with good energy-saving properties and is widely used in various fields (18, 19). The biological benefits of magnetic fields have been explored for decades. Gerencser et al. (20) discovered that magnetic fields can inhibit the growth of some bacteria. Fojt et al. (21) reported a significant decrease in the number and denitrification activity of P. denitrificans exposed to magnetic fields of strength 0–100 Gs. Notably, Shikata et al. (22) applied an oscillating magnetic field (1 Gs) to reduce crystal damage during thawing and freezing to enhance the survival of rat bone marrow mesenchymal stem cells. With these findings, magnetic fields have come to the attention of researchers in the food industry. The quality of cucumbers treated with SMF (70 Gs) was improved during cold storage (23). Additionally, magnetic fields have been widely used in the freezing process and promoted to practical applications, such as Cell Alive System (CAS) refrigerators that are now sold (24, 25). Okuda et al. (1) improved the quality of muscle tissue by freezing mackerel in a CAS freezer with a weak oscillating magnetic field (<20 Gs). In general, there has been an increasing interest recently in the study of magnetic fields in the food industry. At this point, its successful application in the meat freezing process and the control effect of magnetic field on ice crystals provide us with good ideas (26).

According to our investigation, the application of magnetic fields in food thawing has not been reported. Thus, this paper studies the impact of magnetic field assisted thawing (MAT) on meat quality and the objective of this study is to evaluate the impact of static magnetic field (SMF) treatment on thawing characteristics, physicochemical quality and microstructure of frozen beef tenderloin.

## Materials and Methods

### Materials

Fresh beef tenderloin was selected from the large farmers’ market nearest to the laboratory. The beef was provided from a commercial abattoir that processes the cattle of the same breed and similar weight. Excess external fat and connective tissue were removed firstly, followed by cutting the fresh tenderloin perpendicular to the fiber direction into steaks of 50 mm × 30 mm × 20 mm, each weighing 50 ± 1 g. The beef samples were put into clean airtight bags and frozen for 1 week at −20^°^C in a deep low temperature refrigerator (DW-HW50, MeiLing, Hefei, China), waiting for the subsequent thawing experiment. All the utensils used in the beef sample preparation were cleaned with alcohol.

All chemicals (analytical grade) were purchased from Hefei Yuanen Biotechnology Co., Ltd. (Hefei, China).

### Static Magnetic Field Experimental Apparatus and Its Characterization

**FIGURE 1:**
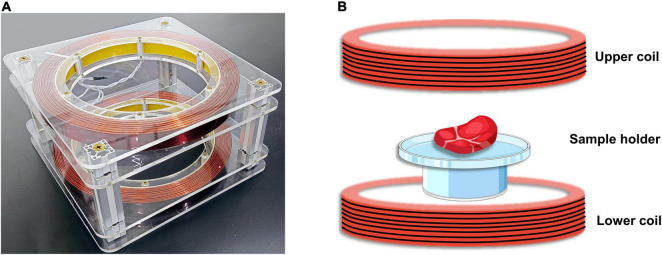
Static magnetic field (SMF) experimental apparatus. **(A)** The physical diagram of the experimental equipment. **(B)** Schematic diagram of magnetic field assisted thawing (MAT) experiment of beef samples.

The static magnetic field experimental apparatus of this research is shown in Figure 1. A pair of Helmholtz coils was used to generate the uniform magnetic field used, with an average coil radius: 15 cm, coil spacing: 15 cm, and number of turns per coil: 168. The pair of Helmholtz coils was placed horizontally in the processing room. The pair of Helmholtz coils was then connected to a DC power supply for energization. By adjusting the power supply, a uniform magnetic field of different strengths was generated in the central region of the coil, whose direction was from top to bottom. The magnetic field intensity was measured with a Gauss meter (GM500, TINDUN Co., Ltd., Shanghai, China).

The schematic diagram of the experiment of magnetic field-assisted thawing of samples is shown in Figure 1B. In order to prove that beef samples on the tray are subjected to uniform magnetic field strength, the magnetic field strength at the center point E and the four vertices at the edge of the tray (points A, B, C, and D) were measured. Besides, the power supply current of the Helmholtz coil was adjusted constantly and the trend of the magnetic field strength with the current was measured. Moreover, to assess the stability of the magnetic field during operation, the intensity at the center of the tray was measured every 10 min.

### Simulation of Static Magnetic Field Experimental Apparatus

Three-dimensional simulations were performed using COMSOL software (v. 5.4, COMSOLAB, Stockholm, Sweden) and AC/DC modules, ignoring non-magnetic materials such as acrylic sheet frames and polyethylene supporting parts on a physical foundation. A magnetic insulation boundary region was established based on the coil shape, and local mesh refinement was applied to the critical regions found (e.g., the inside of the coil) to improve the simulation results. As a result of the simulation, the magnetic field direction distribution of the Helmholtz coil can be obtained, which allows a more intuitive view of the uniform magnetic field generated by the coil.

### Thawing Process

Thawing experiments were performed in the identical processing room, where temperature remained stable. Besides, the air flow in the processing room was negligible and its air velocity was near zero. These frozen steaks were randomly assigned for thawing in a room temperature environment (RT, 24 ± 1^°^C) and magnetic field assisted thawing (MAT, 24 ± 1^°^C), neither of which was affected by any factor other than the presence or absence of static magnetic field during thawing. During thawing, the RT and MAT samples were placed in the optimal magnetic field—the center of the coil. Samples thawed in a room temperature environment were used as controls. The RT samples were thawed when the coil was not applied with supply current and no SMF was present inside. Samples thawed under SMF were classified into five groups defined as MAT-10, MAT-20, MAT-30, MAT-40, and MAT-50 depending on the magnetic field intensity (10, 20, 30, 40, and 50 Gs, respectively). After thawing, the indicators of the samples were detected immediately (thawing loss, cooking loss, color value, TBARS, shear force, and protein content). Meanwhile, some tissues were removed and immersed in fixative and stored at 4^°^C for processing (light microscopy observation, scanning electron microscopy and transmission electron microscopy analysis). To determine each indicator, all experiments were performed at least three times. In addition, a digital thermometer (Model TCP-8X, Huipu, Hangzhou, China) was plugged in the center of the samples, and the insertion port was created at the geometric center of the sample by a microscalpel. The current temperature was output for every 60 s during thawing process.

### Thawing Loss and Cooking Loss

The thawing loss was calculated from the difference in mass of the sample before (*m*_0_) and after (*m*_1_) thawing, which was defined by Equation (1) as follows.


(1) $$\mathrm{Thawing}\mathrm{loss}\left(\%\right)=\frac{m_{0}-m_{1}}{m_{0}}100\%$$

The thawed samples were placed in clean sealed bags and steamed in a 85^°^C water bath. After the core temperature reaches 75^°^C, took it out and wiped off excess water on the surface immediately. We used a digital temperature recorder to measure the core temperature of beef samples (Model TCP-8X, Huipu Instrument Co., Ltd, Hangzhou, China). The cooking loss was calculated from the difference in mass between raw (*m*_*a*_) and steamed samples (*m*_*b*_), which was defined by Equation (2) as follows.


(2) $$\mathrm{Cooking}\mathrm{loss}\left(\%\right)=\frac{m_{a}-m_{b}}{m_{a}}100\%$$

### Color Measurement

The method of color measurement referred to Palmeri et al. (27). Color parameters of RT and MAT samples were measured using a colorimeter (CR-300, Konica Minolta, Tokyo, Japan). To ensure the accuracy of the data, the colorimeter was calibrated with a white standard plate before use. Beef color was expressed in lightness (L^∗^), redness (a^∗^), and yellowness (b^∗^) values by using the instrument’s CIE Lab system.

### Lipid Oxidation

According to the procedure of Sobral et al., (28), thiobarbituric acid reactive substances (TBARS) method was used to measure lipid oxidation. The TBARS value was defined as the number of milligrams of malondialdehyde per kilogram of beef sample (mg MDA/kg) and was used to express the degree of lipid oxidation. A total of 5 g meat samples were homogenized with 25 mL of 7.5% trichloroacetic acid, followed by filtration of the homogenate. A certain amount of thiobarbituric acid solution was added to react. Then the mixture was steamed in a 100^°^C water bath for 45 min. After cooling, absorbance values were recorded at 532 nm using a spectrophotometer (U-5100, HITACHI, Tokyo, Japan).

### Shear Force Values Measurement

The shear force values were determined using a TA-XT plus texture analyzer (Stable Micro Systems, United Kingdom) with a BS model cutterhead. The travel speed of the cutter head was set at 2 mm/s and the forward distance was 30 mm. Six core samples (2 × 1 × 1 cm) were taken from each experimental group of beef and placed on the apparatus platform, followed by cutting these samples perpendicular to the muscle fibers. The maximum force value recorded by the instrument to cut the beef sample was shear force.

### Water-Soluble Protein

The method of water-soluble protein referred to Li et al. (29). A total of 5 g meat samples were homogenized with 45 mL of distilled water followed by centrifugation at 8,000 g for 10 min. The supernatant and the biuret reagent were mixed and left to stand for 15 min. Then, the spectrophotometer (U-5100, HITACHI, Tokyo, Japan) was used to measure the absorbance of the mixture at 540 nm. The absorbance was compared with the standard curve for the determination of bovine serum albumin to obtain the protein concentration of the sample.

### Light Microscopy Observation

By observing micrographs, the effect of SMF on ice recrystallization during thawing and the role of MAT on the structure of muscle fibers can be evaluated more visually. A total of 3 g of beef samples were removed and soaked in 4% paraformaldehyde for 48 h. Immediately afterward the meat samples were cut into (perpendicular to the muscle fibers) thin slices for hematoxylin-eosin (HE) staining. After fixing the treated sample tissues on slides, the state of the muscle structures was observed at 100 × magnification using an Olympus IX optical microscope (Nikon, Tokyo, Japan). In addition, we analyzed the image results to determine the area and interstitial space of muscle fibers by using Image-Pro Plus 6.0 software.

### Scanning Electron Microscopy Analysis

The method of scanning electron microscopy (SEM) was referenced to the steps of Cao et al., (30). A scalpel was used to remove beef samples (2 × 2 × 1 mm) from each experimental group, followed by fixation with 2.5% glutaraldehyde for 12 h and rinsing with phosphate buffer for 15 min. The samples were sequentially dehydrated in gradients of 30, 50, 70, 80, 90, and 100% alcohol concentration. The dried samples were mounted on a bronze stub and sputtered with gold (Cressington, Watford, United Kingdom). Then, a ZEISS GeminiSEM 300 scanning electron microscope was used to observe the fiber cross section of the sample.

### Transmission Electron Microscopy Analysis

The method of transmission electron microscopy (TEM) was referenced to the steps of Kahraman et al., (31). A scalpel was used to remove beef samples (3 × 1 × 1 mm) from each experimental group, followed by fixation with 2.5% glutaraldehyde for 24 h. The samples were washed 4 times with PBS and fixed in 1% osmium tetroxide for 2 h. The strips were then washed with PBS and dehydrated in a sequential gradient with 50, 70, 90, and 100% concentrations of ethanol. Immediately, the samples were permeabilized, embedded, sectioned and stained. Then, a Thermoscientific Talos L120C G2 transmission electron microscope was used to observe the beef samples.

### Statistical Analysis

All data were expressed as mean values mean ± *SD*. One-way ANOVA with Tukey multiple comparisons was used to determine the significance of differences, and the confidence level was set at 95 %. A *p*-value less than 0.05 was considered statistically significant. ns means there was no significant difference between them, ^∗^indicates *P* < 0.05, ^∗∗^indicates *P* < 0.01, ^∗∗∗^indicates *P* < 0.001.

## Results and Discussion

### Simulation and Characterization of Static Magnetic Field Experimental Apparatus

**FIGURE 2:**
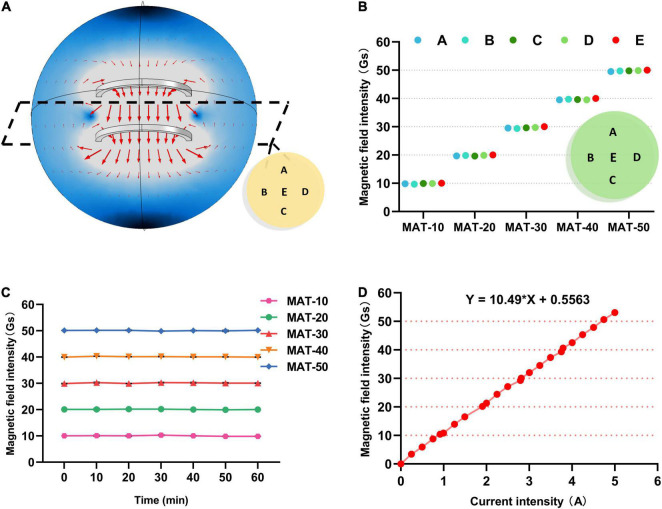
Simulation and Characterization of SMF experimental apparatus. **(A)** The general law of the magnetic induction direction distribution in the coil. **(B)** The magnetic field intensity value of each point A, B, C, D, E on the sample plate of each group in the MAT treatment group. **(C)** The magnetic field intensity value at the center of the tray (point E) during MAT experiments. **(D)** The relationship between the magnetic induction intensity of the coil and the supply current.

The general pattern of the magnetic field direction distribution in the coil is shown in Figure 2. Magnetic induction lines inside the coil were always vertical downward, and no obvious deviation in the direction (Figure 2A). When the supply current of the coil was adjusted so that the magnetic induction intensity in the center of the coil was different, magnetic field distribution in the coil was also approximately similar.

The magnetic induction strength of the coil and the current were linearly related by continuously adjusting the power supply current of the coil (Figure 2D). Then, the supply current of the coil was adjusted to achieve the magnetic field strength of each group for the experiment. Meanwhile, the magnetic field intensity at each point of the tray was also measured, and it was concluded that the beef samples were in a uniform magnetic field (Figure 2B). Moreover, the intensity of the magnetic field inside the Helmholtz coil remained constant over long periods of operation, demonstrating the stability of the coil (Figure 2C).

### Effect of the Magnetic Field Assisted Thawing on the Thawing Rate

**FIGURE 3:**
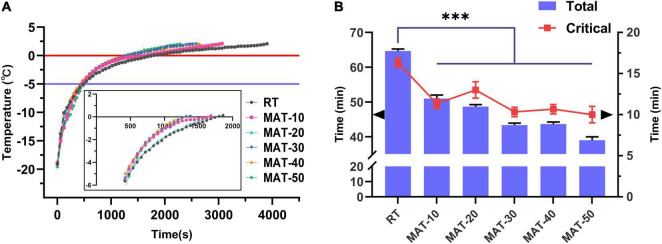
Thawing curves of frozen beef with different thawing treatments. **(A)** Evolution of temperature at the center of beef samples during RT and MAT thawing experiments. **(B)** Total thawing time and critical time of frozen beef with different thawing treatments. Critical time, the time of passing the maximum ice crystal formation zone. ^∗∗∗^ indicates *P* < 0.001.

The center temperature of the beef tenderloin was measured during thawing. The temperature change curve of beef during thawing is shown in Figure 3. In the field of food preservation, the temperature range of −5 to 0^°^C is considered to be the maximum ice crystal formation zone (32). The total time of thawing and the time to pass the maximum ice crystal formation zone (Critical time) of the MAT treatment group were significantly lower than those of the control group. The shortest total thawing time was with MAT-50 which was 40% lower than the control, followed by MAT-30 and MAT-40. This shows that MAT helps shorten the thawing process of beef. Similarly, RT took 16 min to pass the temperature range of −5 to 0^°^C, while the time of MAT was significantly shortened, of which MAT-50 only took 10 min. Since magnetic field inhibited the formation of large ice crystals, samples treated with SMF might require less heat absorption to melt the ice crystals (22, 33). The conclusion obtained in total and critical time of thawed samples proved the previous analysis that SMF may reduce the growth of ice crystals during recrystallization, and the effect of tiny ice crystals can significantly accelerate thawing (Figure 3B).

### Effect of the Magnetic Field Assisted Thawing on Thawing Loss and Cooking Loss

**FIGURE 4:**
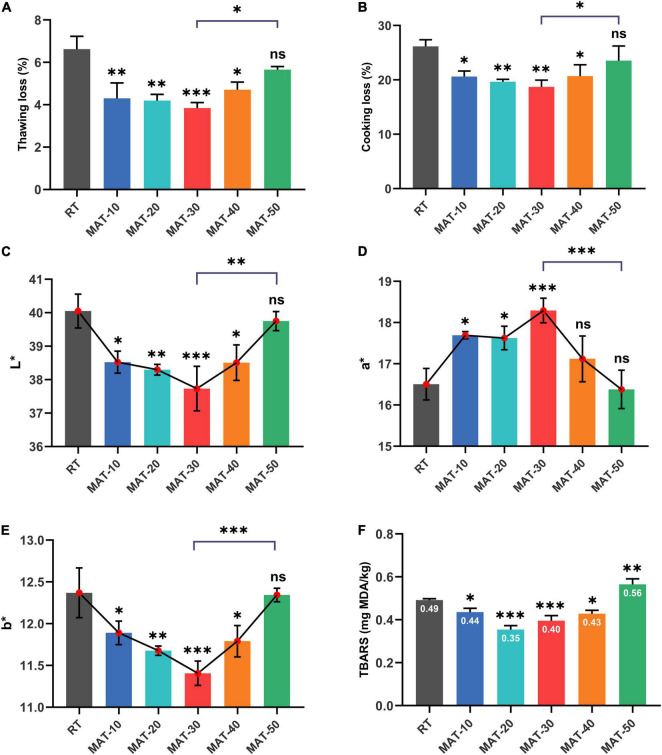
The quality parameters of frozen beef with different thawing treatments. **(A)** Thawing loss. **(B)** Cooking loss. **(C)** L* values. **(D)** a* values. **(E)** b* values. **(F)** TBARS values. ^∗^ indicates *P* < 0.05, ^∗∗^ indicates *P* < 0.01, ^∗∗∗^ indicates *P* < 0.001.

**FIGURE 5:**
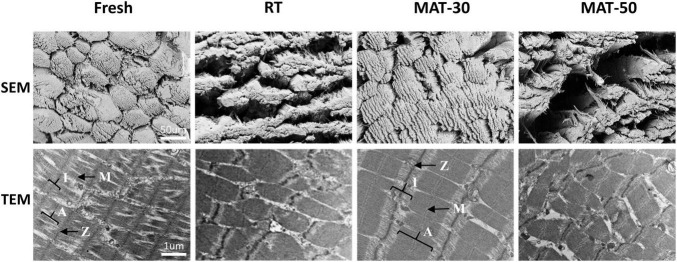
Microstructure of frozen beef with different thawing treatments.

These indicators were measured immediately after the sample was thawed to avoid any excess reaction or volume change. Thawing loss and cooking loss are key indicators that affect meat quality (3, 34). As described in Figure 4, these parameters of thawed beef were lower when magnetic field intensity was 30Gs, but increased rapidly when the magnetic field intensity (MAT-40 and MAT-50) was further enhanced. The thawing loss and cooking loss of MAT-30 were the smallest (3.8 and 18.7%), which were 42 and 29% lower than RT (6.6 and 26.2%), respectively. The dripping water contains substances and water exuded from the cells destroyed by the freezing and thawing process (35). Figure 4A showed that the thawing loss of the RT was significantly greater than that of the MAT group, which indicated that more protein was lost with the thawing drip of RT. As a result, these losses could affect the quality of beef in terms of taste and texture, while the exudate also provides nutrients for bacterial to multiply. Water holding capacity (WHC) is usually expressed by thawing loss and cooking loss, and is a key parameter to measure meat quality (36). Thawing time, water reabsorption, size of ice crystals, structural integrity of muscle tissue and WHC are factors responsible for water loss during food thawing (37). These results indicated that compared with RT, samples treated with appropriate magnetic field intensity (MAT-10, MAT-20, and MAT-30) could significantly increase WHC and reduce nutrient loss after thawing. However, when the magnetic field intensity (MAT-40 and MAT-50) is further enhanced, the myofibril structure may suffer severe damage, causing myofibroblasts to lose a lot of water during thawing and cooking (Figure 5).

### Effect of the Magnetic Field Assisted Thawing on the Color and Thiobarbituric Acid Reactive Substances

Consumers’ willingness to purchase is largely influenced by the color of the meat (3). Numerous previous studies on meat have demonstrated that consumers generally believe that pleasant colors will be fresher, higher quality and better flavor (38, 39). The surface color of beef under different thawing conditions was assessed by lightness (L^∗^), redness (a^∗^), and yellowness (b^∗^) (Figure 4). RT sample had the highest L^∗^ and b^∗^ values (40.1 and 12.4), which were higher than those of the lowest MAT-30 sample (37.7 and 11.4) by about 6 and 9%, respectively. The a^∗^ value of the MAT-30 sample was the highest (18.3), which was nearly 11% higher than the RT (16.5). The L^∗^ values of MAT-10, MAT-20, MAT-30, and MAT-40 samples were significantly lower than RT samples, but there was no difference between RT and MAT-50. The variation of the b^∗^ values of the sample were similar to that of the L^∗^ values. As for samples (MAT-10, MAT-20, MAT-30) treated with suitable magnetic field, the L^∗^ and b^∗^ values decreased as the magnetic field intensity increased. However, the L^∗^ and b^∗^ values increased when the magnetic field intensity (MAT-40 and MAT-50) was further enhanced. The change of the a^∗^ values were just the opposite.

The increased L^∗^ values of meat after thawing was harmful, which indicated that free water diffused to the surface and reflected more light (40). A large amount of thawed dripping water might cause greater light reflections in RT sample. Also, a higher strength magnetic field increased the L^∗^ value of the muscle as more drops was removed from the cell. The mechanism that MAT causes the a^∗^ value of meat to change may be that the value correlates with the protein content of the meat (e.g., myoglobin). Loss of cell contents during thawing may result in protein loss and denaturation, which can decrease a^∗^ value of beef. Moreover, some studies have shown that the generation of extracellular gaps is the main cause of the increase of the L^∗^ values and the decrease of the a^∗^ values (41). Our subsequent observation of the microstructure supports this view. In addition, lipid oxidation was the main cause of increased b^∗^ values (42), and the magnitude of lipid oxidation in different treatment samples seemed to be related to the b^∗^ values (Figure 4F). The a^∗^ values (redness) provided consumers with the best judgment for accepting beef color, and when the a^∗^ value was higher, the quality was considered better (43). The above result indicated that MAT could prevent beef from discoloration compared to RT, thus increasing consumer acceptance.

TBARS values of each group of thawed beef samples were shown in Figure 4F. The TBARS values of MAT-10 to MAT-40 samples treated with static magnetic field were significantly lower than RT samples. The results indicated that the appropriate magnetic field intensity could restrain lipid oxidation to a certain extent. However, TBARS values of MAT-50 treated with excessive magnetic field intensity were significantly greater than those of RT, which may be due to cell damage and subsequent production of prooxidants that caused lipid oxidation (44).

### Effect of the Magnetic Field Assisted Thawing on the Shear Force and Protein Content

**FIGURE 6:**
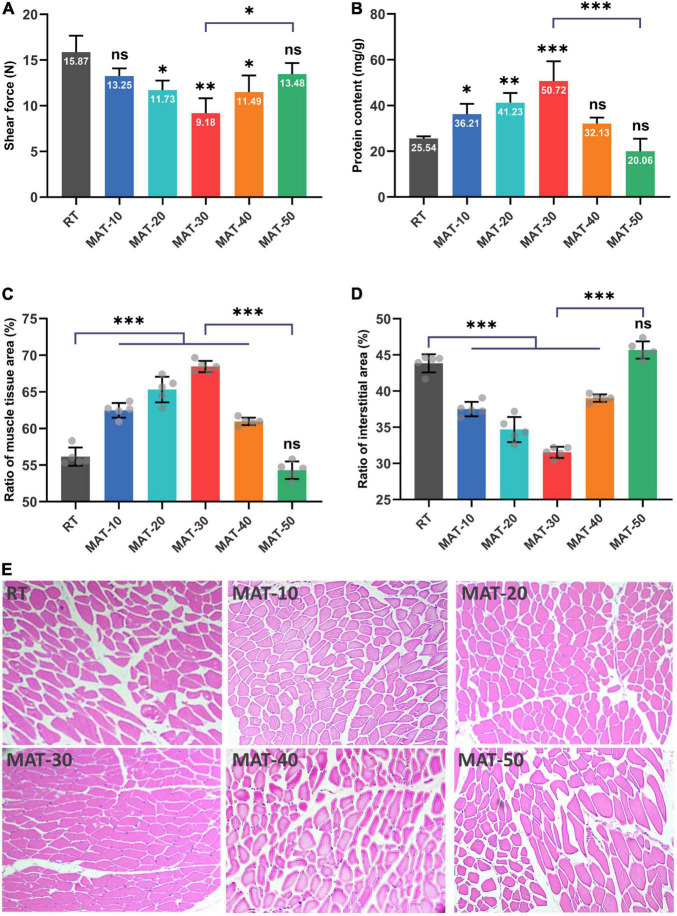
Physicochemical quality of frozen beef with different thawing treatments. **(A)** Shear force value. **(B)** Water-soluble protein content. **(C)** The ratio of muscle tissue area. **(D)** The ratio of the interstitial area in each muscle tissue. **(E)** Structure of muscle fiber. ^∗^ indicates *P* < 0.05, ^∗∗^ indicates *P* < 0.01, ^∗∗∗^ indicates *P* < 0.001.

Tenderness is a key parameter reflecting the texture of meat, and shear force is used to determine this index (34). It can be seen from Figure 6A that the shear force value of RT was significantly greater than that of samples treated with magnetic field. The MAT-30 group showed the lowest shear force and effectively reduced the deterioration of tenderness of frozen beef tenderloin after thawing, which demonstrated the protective effect of MAT on the texture of beef. The magnitude of the shear force depends on the moisture content and myofibril structure of meat. The higher the water holding capacity, the greater the tenderness (45). The appropriate magnetic field intensity could improve the structure of beef samples resulting in smaller shear values. It is notable that excessive magnetic field intensity did not significantly reduce the shear force value of beef samples. The shear force value of beef samples became larger under higher magnetic field intensity probably due to the increased water loss during thawing. Previous studies have indicated that minor damage to muscle fibers contributes to a reduction in shear force as well, but excessive damage increases shear force because of decreased water holding capacity (46). In addition, the fiber structure of the MAT clearly had better integrity than that of the RT group (Figures 5, 6). The above results showed that beef samples thawed at appropriate magnetic field intensity had better texture compared to the RT group.

The main component of water-soluble protein is sarcoplasmic protein, which is a soluble protein in beef tenderloin. It provides insight into the integrity of cells in beef samples, as ice crystals formed during freezing and thawing can puncture cells, resulting in the outflow of water-soluble proteins and other contents (29). It can be seen from Figure 6B that the protein content of MAT-10 to MAT-30 samples treated with static magnetic field were higher than that of RT. However, the MAT-40 and MAT-50 samples treated with excessive magnetic field intensity were not significantly different from the control group. The protein content of the MAT-30 sample treated with appropriate magnetic field intensity was significantly higher than that of RT. Protein loss may be caused by cells suffering severe damage from recrystallization phenomena during thawing, causing loss of cell contents. Since the ice crystals formed by thawing under SMF are finer, the damage to the cells becomes smaller and therefore the loss of cell contents is reduced.

### Structure of Muscle Fiber

To evaluate the muscle damage caused by the freeze-thaw process, beef samples from different thawing treatments were subjected to histological analysis (Figure 6E). The muscle fiber of the RT samples was severely damaged, and the boundaries between adjacent fibers were blurred. Samples treated with appropriate magnetic field intensity (MAT-10, MAT-20, and MAT-30) had particularly uniform distribution of muscle fibers, along with narrow extracellular gaps between tissues and a relatively regular shape of muscle tissue in cross-section. When the magnetic field intensity (MAT-40 and MAT-50) was further enhanced, the muscle fiber distribution began to disperse, and the extracellular space between the muscle tissues increased. Excessive magnetic field intensity (MAT-50) could also cause muscle fiber damage like the control group.

We can see from Figure 6E that there were significant structural differences between MAT-10 to MAT-40 muscles treated with static magnetic field and the RT muscles of the control group. In contrast, MAT-50 exhibited the same expansion of extracellular space and disorganized myofiber arrangement as RT. Significant difference in muscle tissue area ratio between MAT-10 to MAT-40 samples treated with static magnetic field and the RT samples, but the MAT-50 sample was not significantly higher than the RT sample (Figure 6C). Similarly, there was a significant difference in the interstitial area ratio between the MAT-10 to MAT-40 samples treated with the static magnetic field and the RT samples, but there was no significant difference between MAT-50 and RT (Figure 6D). The proportion of MAT-30 muscle fibers was the largest and the gap between muscle fibers was the smallest. Therefore, the muscle fibers of MAT-30 suffered the least damage from ice recrystallization during thawing, reducing secondary damage from ice crystals and maintaining the quality of beef samples. In addition, samples treated with excessive magnetic field intensity may cause cell atrophy due to a large amount of water loss, resulting in damage to the muscle fiber structure. Myofibrillar structural damage during the thawing process also reduced the ability of myofibrils to retain water through the capillary force between the thick and thin filaments (47).

### Microstructures

The microstructure of meat is closely related to its quality (e.g., WHC and texture) (11). From microstructure of the cross-section of muscle tissue, it can be clearly seen that the muscle fiber structure of fresh beef was complete, full and tightly arranged (Figure 5). Compared to fresh state, muscle fibers of the control group and the magnetic field treated group (MAT-30 and MAT-50) were elongated and the structural integrity of fiber bundles suffered damage. These images demonstrate that meat samples processed by freezing and thawing inevitably cause some damage to the microstructure of the muscle (48). The gaps between the fiber bundles in the RT group were severely enlarged, and the endomysium was also severely torn. As for samples (MAT-30) treated with suitable magnetic field, the fiber bundles were significantly more tightly arranged and the muscle microstructure was more intact. However, excessive magnetic field intensity (MAT-50) could produce large gaps between muscle fibers as in the control group. These gaps will form water channels (49), through which water leaks out, further creating drip losses. The lax structure leads to a decrease in the WHC of the muscle, which exactly corroborates the above results of thawing losses.

The ultrastructure of the muscles taken by TEM under different thawing methods can be seen in Figure 5. Muscle fibers are made up of myofibrils, which are made up of numerous sarcomeres. The sarcomere is the area between two adjacent Z-lines, which is composed of one A-belt, one M-line, and two halves of the I-zone (50). For fresh beef, the myofibrils were shown to have a complete sarcomere structure with clear and complete the M-line, Z-line, A-belt, and I-zone in myofibrils. RT samples exhibited significant disruption of sarcomere structure and weakening of the M-line, which may be due to the formation of numerous ice crystals during thawing and large-size ice crystals produced during the process of slow thawing. However, the ultrastructure of MAT-30 sample was almost the same as the fresh state, suggesting that appropriate magnetic field intensity (MAT-30) could minimize the microstructure damage caused by recrystallization during thawing. As for samples (MAT-50) treated with excessive magnetic field intensity, the myofibrils exhibited the Z-line disintegration and sarcomere dislocation. Therefore, compared to RT and MAT-50, muscle fibers of MAT-30 were neatly aligned, and the sarcomere structure was more complete and well defined. The contraction of the sarcomere is accompanied by the water being squeezed out, which in turn produces dripping (51). The deterioration of the tenderness of meat is also mainly caused by sarcomere shortening and cracking of muscle fibers (52). From the SEM and TEM images, it can be seen that the damage of MAT-30 to the muscle microstructure is significantly less than other thawing treatments, which is consistent with the reduction of thawing loss and higher texture quality (Figures 4, 6).

### Analysis of Underlying Mechanism

**FIGURE 7:**
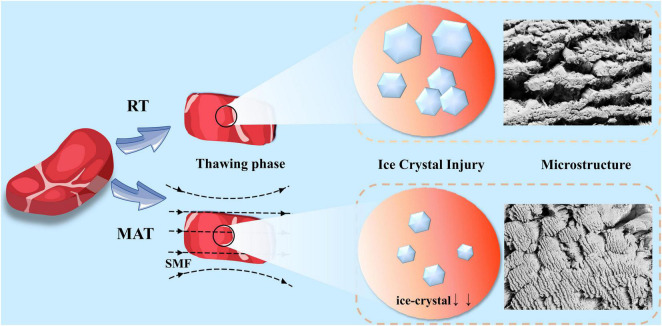
Schematic diagram of the underlying mechanism of MAT.

The underlying mechanism of MAT is shown in Figure 7. Combined with light microscopic observation of muscle fibers and microstructure images, the effect of SMF on ice crystal formation during sample recrystallization can be found. The magnetic field acts on water molecules by aligning the electron spin and nuclear spin of the atom in the direction of the magnetic field. When it works, the hydrogen nuclei can be like a small bar magnet and aligned with the external magnetic field (19, 53). When frozen samples are thawed under SMF, the spin nucleus will exhibit precession movement around the direction of the applied magnetic field which can strengthen the thermal vibration of the hydrogen nucleus and the heat conduction of the entire product, further increase the thawing speed, thereby reducing the formation and duration of recrystallization (shortened critical time). In addition, the water molecules will be subjected to migration by external magnetic fields, which will prevent the water molecules from clustering and destroy the large water clusters to form smaller ones, thus inhibiting crystallization (33, 54, 55). Appropriate magnetic field strength treatment inhibited the clusters of water molecules in the thawed samples, which in turn inhibited the generation of large ice crystals during the thawing process, allowing more fine ice crystals to be evenly distributed in the muscles, improving the thawing speed while reducing thawing loss and damage to muscle structure, thus improving the quality of thawed beef. Although excessive magnetic field strength can greatly improve the thawing speed, it may result in the partially melted water being not able to return to the intracellular space after thawing, which may increase thawing drips and protein loss, and ultimately lead to poorer thawed beef quality. In addition, a large amount of water loss could cause cell atrophy causing damage to the muscle fiber structure (47, 50).

## Conclusion

The current research mainly focused on the impact of MAT on thawing characteristics, post-thawing quality and microstructure of frozen beef tenderloin. Under SMF, thawing process of beef was effectively shortened. Furthermore, by protecting the microstructure of the muscle, MAT significantly reduced thawing loss, TBARS values, cooking loss and shear force, and increased a^∗^ values and protein content. In short, the use of MAT with appropriate magnetic field strength can maintain the physicochemical quality of beef to a certain extent, and reduce the damage of ice crystals to muscle fibers. The findings of this study demonstrated the potential application of SMF in meat thawing technology, which will facilitate the effective maintenance of the “fresh” quality of meat and provide a theoretical basis for the application of novel thawing technologies.

## Data Availability Statement

The original contributions presented in the study are included in the article/supplementary material, further inquiries can be directed to the corresponding author/s.

## Author Contributions

JJ: conceptualization, methodology, investigation, formal analysis, and writing—original draft. LZ and JY: investigation and validation. YC and ZC: supervision, writing—review, and editing. GZ: supervision, conceptualization, funding acquisition, writing—review, and editing.

## Conflict of Interest

The authors declare that the research was conducted in the absence of any commercial or financial relationships that could be construed as a potential conflict of interest.

## Publisher’s Note

All claims expressed in this article are solely those of the authors and do not necessarily represent those of their affiliated organizations, or those of the publisher, the editors and the reviewers. Any product that may be evaluated in this article, or claim that may be made by its manufacturer, is not guaranteed or endorsed by the publisher.
